# Supplementary material for: Heterogeneity of Alzheimer’s disease identified by neuropsychological test profiling
Source: PLoS One. 2023 Oct 5;18(10):e0292527. doi: 10.1371/journal.pone.0292527 (PMC10553816; doi:10.1371/journal.pone.0292527)
Supplement: S1 Table — (DOCX) [file pone.0292527.s001.docx]

**Heterogeneity of Alzheimer's disease identified by neuropsychological test profiling**

**Supporting Information**

**S1 Table**. The Cognitive Abilities Screening Instrument (CASI) instrument, version Chinese 2.0, total scores ranging from 0 to 100 (1,2).

| **Cognitive Domain** | **Item** | **Maximum Score** |
| --- | --- | --- |
| **Orientation** | - How old are you? - What is today's date? - What day of the week is today? - Is it morning, noon, afternoon or evening?^*,a^ - Is this place a store, a hospital (clinic), or home? - What city/district are we in? | 18 |
| **Long-term memory** | - How many months are there in a year? - What month is the Chinese New Year? - How many minutes are there in an hour? (or How many days are there in a year?) - In what direction does the sun set? - On what festival is moon cake eaten? (Mid-Autumn festival, August festival)^*,b^ | 10 |
| **Attention** | - Repeat three words immediately. - Repeat two sentences immediately^*,c^. | 8 |
| **Short-term memory** | - Recall the three words from the "Attention" section twice^*,d^ (with an approximately 10-minute gap) - Recall five objects from the "Language" section. | 12 |
| **Language** | - What animals have 4 legs? - Follow written command: "Close your eyes". - Follow verbal command: "Take this paper with your left (right) hand, fold it in half, and hand it back to me". - Write the five words "person, father, mother, male, female" in Chinese^*,e^ - Recall parts of the body (forehead, chin, shoulder, palm, thumb) - Recall name of objects (spoon, coin, toothbrush, key, comb) | 20 |
| **Concentration** | - Digit span backwards - Serial subtraction 100 - 3^*,f^ | 10 |
| **Abstraction & Judgment** | - Tell the similarity between i) fish and shrimp, ii) a table and a chair, iii) hand and foot. - What actions would you take if you saw your neighbor's home catching fire? - What actions would you take if you lost a borrowed umbrella? - If you see someone else's lost ID card on the road, what will you do? | 12 |
| **Visuospatial ability** | - Draw a circle, a diamond, and double pentagons. | 10 |

Note: Items were listed based on their cognitive domains, not the order in which they are tested on participants.

^*^ Items modified from the original English version to accommodate Chinese elders with limited educational backgrounds.

^a^ Original version: ask participants about the season. Modified because the Chinese version was initially conducted in Kinmen (Taiwan), an island with indistinct seasonal changes.

^b^ Original version: ask for date of birth. Modified because many Chinese elders were born when official birth records were not often documented.

^c^ Original version: ask participants to repeat "This yellow *circle* is heavier than blue *square*", which was modified to "This yellow *cup* is heavier than red *rice bowl*", because "*circle*" and "*square*" were abstract terms considered rarely used in nonliterate Chinese elders' lives.

^d^ One of the three words was modified from "*honesty*" (considered an uncommon word for poorly educated people) to "*child*".

^e^ Reading and writing accounted for 4 points in the original version, but only 2 points in the Chinese version, and the writings included very simple Chinese characters. Scores are instead weighted to the naming ability.

^f^ Original version: serial subtraction of 3s from 100. Chinese version: ask participants how much money they would have left if they had $100 and spent $3, and so on.

**References**

1. Teng EL, Hasegawa K, Homma A, Imai Y, Larson E, Graves A, et al. The Cognitive Abilities Screening Instrument (CASI): a practical test for cross-cultural epidemiological studies of dementia. Int Psychogeriatr. 1994;6(1):45–58; discussion 62.

2. Yeo G, Thompson DG. Ethnicity and Dementias. Taylor & Francis; 2014. 288 p.
