# Supplementary figures and images for: Heterogeneity of Alzheimer’s disease identified by neuropsychological test profiling
Source: PLoS One. 2023 Oct 5;18(10):e0292527. doi: 10.1371/journal.pone.0292527 (PMC10553816; doi:10.1371/journal.pone.0292527)

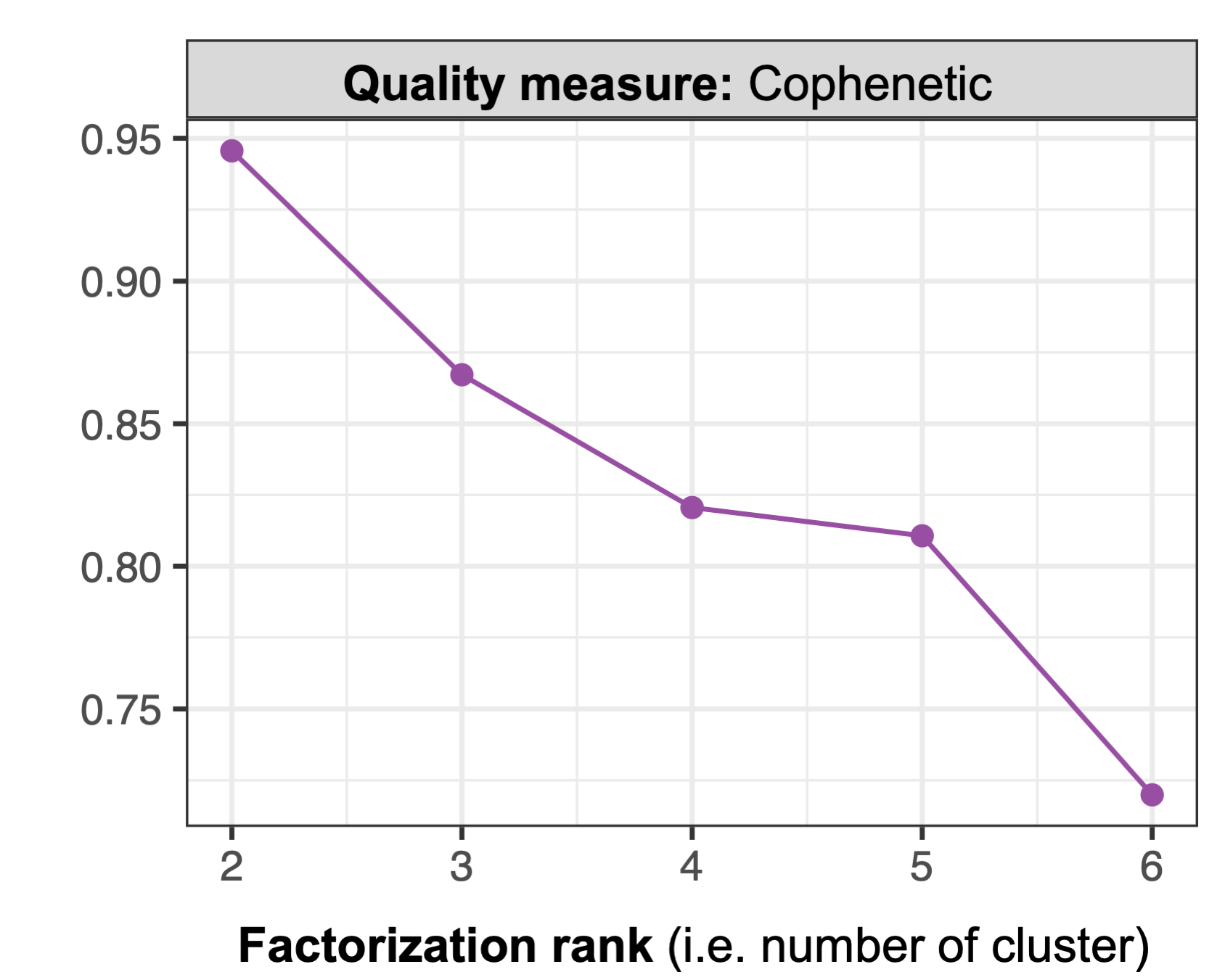

Supplement: S1 Fig — (TIFF) [file pone.0292527.s002.tiff]
