# Supplementary material for: Heterogeneity of Alzheimer’s disease identified by neuropsychological test profiling
Source: PLoS One. 2023 Oct 5;18(10):e0292527. doi: 10.1371/journal.pone.0292527 (PMC10553816; doi:10.1371/journal.pone.0292527)
Supplement: Attachment — Submitted filename: Response to Reviewers (PONE-D-23-03358).docx [file pone.0292527.s003.docx]

PONE-D-23-03358

Heterogeneity of Alzheimer's disease identified by neuropsychological test profiling

PLOS ONE

**REVIEWER #1 (Comments to the Authors)**

In the submitted manuscript, the authors aimed to investigate cognitive heterogeneity in Alzheimer’s Disease (AD), one of the open questions in the literature on dementia and its cognitive correlates.

To this end, they enrolled a sample of patients (N=112) with a clinical diagnosis of mild-moderate AD. All patients were subjected to a neurocognitive assessment (MMSE, CASI, CDR) and a subset of them also underwent a neuroimaging acquisition session (either CT or MRI).

The authors applied a data-driven approach (nonnegative matrix factorization) to identify cognitive clusters in their sample of patients and were able to identify two clusters characterized by predominant memory (62.5%) vs. non-memory (37.5%) deficits. Patients in the memory group had also a worse cognitive performance at baseline.

Overall, the paper is well-written, has several methodological strengths (analytic methods are also well described), and deals with a critical issue, namely the heterogeneity of cognitive profiles in AD patients and the need to map such heterogeneity to promote individualized treatments.

We are thankful to the reviewer for their very detailed feedback on the manuscript. Addressing their concerns has significantly improved the manuscript, and substantiated the validity of the results.

The following are some minor points that hopefully could contribute to improving the present manuscript before its publication.

- The introduction (and the discussion) would benefit from a wider discussion of literature on clusters in Alzheimer’s disease. Indeed, there is a bunch of studies showing different numbers of clusters, despite some overlap with the results of the present study (e.g., Zangrossi et al., 2021, doi: 10.3233/JAD-210719, Journal of Alzheimer’s Disease; Cappa et al., 2014, Behav Neurol). I believe that a discussion of the present results in the light of previous studies showing different (but somehow matching) results would contribute to the strength and robustness of the present paper.

R1.1. We thank the reviewer for their suggestion. We have expanded the Introduction and Discussion to include references that extended our study results. They read as follows:

"For instance, Scheltens et al. employed a data-driven clustering method on neuropsychological measurements and identified two subgroups of AD with discrete cognitive profiles *(Scheltens et al., 2017; Scheltens et al., 2018)*. One subgroup, the "memory" AD, had lower scores in memory-related tests, more severe hippocampal atrophy, slower disease progression and lower risk of mortality, compared to the remaining "non-memory" group that showed impairment in the language or executive/visuospatial domain. This finding strongly indicates that cognitive heterogeneity could predict individuals who are at greater risk of deterioration in dementia." (line 68-75)

"Previous work by Cappa et al., which employed one of the most comprehensive NP battery, found four hierarchical clustering-derived subtypes, namely the memory, visuospatial/perceptual, perceptual/calculation, and language from a cohort of patients with AD and posterior cortical atrophy *(Cappa et al., 2014)*. Similarly, Zangrossi et al. identified four AD groups – typical, mild, visuospatial, and nonamnestic – that were partly explained by differences in sex and global cognitive functioning *(Zangrossi et al., 2021)*. Scheltens et al. employed latent class analysis and identified eight AD cognitive clusters characterized by discrete neuropsychological performance and disease severity *(Scheltens et al., 2016)*." (line 361-368)

Scheltens NME, Tijms BM, Koene T, Barkhof F, Teunissen CE, Wolfsgruber S, et al. Cognitive subtypes of probable Alzheimer’s disease robustly identified in four cohorts. *Alzheimer’s & Dementia*. 2017;13(11):1226–36.

Scheltens NME, Tijms BM, Heymans MW, Rabinovici GD, Cohn-Sheehy BI, Miller BL, et al. Prominent Non-Memory Deficits in Alzheimer’s Disease Are Associated with Faster Disease Progression. *J Alzheimers Dis*. 2018;65(3):1029–39.

Cappa A, Ciccarelli N, Baldonero E, Martelli M, Silveri MC. Posterior AD-Type Pathology: Cognitive Subtypes Emerging from a Cluster Analysis. *Behavioural Neurology*. 2014:e259358.

Zangrossi A, Montemurro S, Altoè G, Mondini S. Heterogeneity and Factorial Structure in Alzheimer’s Disease: A Cognitive Perspective*. J Alzheimers Dis*. 2021;83(3):1341–51.

Scheltens NME, Galindo-Garre F, Pijnenburg YAL, Vlies AE van der, Smits LL, Koene T, et al. The identification of cognitive subtypes in Alzheimer’s disease dementia using latent class analysis. *J Neurol Neurosurg Psychiatry*. 2016;87(3):235–43.

- Neuroimaging-derived parameters are poorly discussed. They are not entered in the NMF clusterization and in subsequent analyses. Why did not the authors include such measurements in the linear mixed-effect model? If this is not a variable of interest I suggest to clearly state it in the introduction or in the “clinical measurements” paragraph.

R1.2. We thank the reviewer for their thorough reading. They are correct in that neuroimaging variables were not used extensively in this study (only in Table 1, where we compared the medial temporal lobe atrophy (MTA) score and white matter hyperintensities/Fazekas scale between the two subgroups). They were also not included in the linear mixed-effect model as they were only available at baseline examination of the participants, and not during follow-ups.

One alternative is to use neuroimaging software, such as FreeSurfer, to perform cortical parcellation and subcortical segmentation, which will provide a richer dataset in this study. Here we have opted to include the MTA and Fazekas measures, which are visual rating scales that can easily be applied in clinical settings.

We have added these points in the manuscript at line 123-127, 331-333, and 338-341.

- Baseline MMSE: please, discuss why the clustering could not just be interpreted as representing different degrees of disease severity (since there are differences in baseline MMSE).

R1.3. We thank the reviewer for this insight. We agree with the reviewer that heterogeneity can be caused by both disease subtype and disease stage. The field's opinions on this matter has been somewhat mixed. On the one hand, it could be interpreted that the variance identified from subtype analysis is actually driven by different disease stages, thus not true (cognitive) heterogeneity per se *(Habes et al., 2020).* On the other hand, one could argue that temporal and phenotypic heterogeneity are unavoidably intertwined and develop algorithms that take into account both simultaneously, as was done in *Young et al., 2018*.

In our study, we believe that disease stage did not substantially contribute to the cognitive subgroups, because the "age at disease onset" and "duration of onset" did not differ significantly between the two groups (Table 1). One more thing we could do is to validate the cognitive clusters after stratification for disease severity, i.e. split the cohort into two groups, mild and moderate in terms of severity, using the median MMSE/CASI/CDR scores, and repeat the cluster analysis on each group to see if the results would be similar *(Scheltens et al., 2017)*. We elected to not perform this because the original sample size was only n = 112 and splitting the cohort further would likely result in too small sample size. Hence, we have added these arguments to the manuscript at line 316-329.

Habes M, Grothe MJ, Tunc B, McMillan C, Wolk DA, Davatzikos C. Disentangling Heterogeneity in Alzheimer's Disease and Related Dementias Using Data-Driven Methods. *Biol Psychiatry*. 2020;88(1):70-82.

Young AL, Marinescu RV, Oxtoby NP, et al. Uncovering the heterogeneity and temporal complexity of neurodegenerative diseases with Subtype and Stage Inference. *Nat Commun*. 2018;9(1):4273.

Scheltens NME, Tijms BM, Koene T, et al. Cognitive subtypes of probable Alzheimer's disease robustly identified in four cohorts. Alzheimers Dement. 2017;13(11):1226-1236.

- Longitudinal trajectory: please clarify whether patients were followed for 3 (line 241) or 5 years (line 231) and which data were included (e.g., only the first 3 years of follow-up). Moreover, did authors take into account the time distance between timepoints (e.g., see Tondo et al., 2021, Env Res and Pub Health).

R1.4. We sincerely thank the reviewer for their thorough reading and apologize for the confusion.

We would like to clarify that there were three instances where longitudinal data were involved in our study. Firstly, the total number of years of follow-up of each patient was collected and compared between those in the memory and non-memory group (Table 1). Secondly, we visualized the trajectory of disease progression via raw scores of MMSE, CASI, and CDR (Fig 3). Finally, we compared the rate of disease progression between two subgroups using linear mixed effect model analysis (Table 2 and Fig 4).

Below we show the number of participants that had available NP measures throughout the years in our study:

| **TIME** | **Baseline** | **Year 1 (Y1)** | **Y2** | **Y3** | **Y4** | **Y5** | **Y6** | **Y7** | **Y8** |
| --- | --- | --- | --- | --- | --- | --- | --- | --- | --- |
| **NUMBER OF PARTICIPANTS** | 112  (100%) | 83  (74%) | 76  (68%) | 65  (58%) | 43  (38%) | 19  (17%) | 7  (6%) | 3  (2%) | 1  (0.8%) |

For the linear mixed effect model analysis, we only included data within 3 years of follow-up because from the 4th year on the number of participants with available data dropped substantially (n = 43 from 112), which could induce the linear mixed effect model algorithm to fail to converge (i.e. it would not reach a stable solution with maximum likelihood, or participant with only 2 years of follow-up has more missing values than one that was followed for, say, 5 years). For the visualization of disease trajectory using raw scores, however, because there were no statistical analyses involved, we opted to include data of up to 5 years *(Rongve et al., 2016).* We have added the following sentence to clarify this in the manuscript, line 255-259.

"We only included data within three years of follow-up as from the fourth year on the number of participants with available data dropped substantially (n = 43 from 112), which could induce the linear mixed effect model algorithm to fail to converge, i.e. it would not reach a stable solution with maximum likelihood."

Regarding whether we took into account the time distance between timepoints, we believe that our approach and that by *Tondo et al., Env Res and Pub Health 2021* are different. Tondo et al. calculated the "index of progression" based on the formula follow-up MMSE−baseline MMSE/years of follow-up and compared this index between the three 2020, 2019, and 2018-groups using Kruskal–Wallis test. Hence, they were interested in only two timepoints (T0 and T1), which were mostly 1 year apart (e.g. "Follow-up in years" 0.93 ± 0.15 for the 2020 group), and excluded any patients with missing data. In contrast, our study included participants that were followed up for different number of years (e.g. the "memory" group were followed for 3.4 ± 1.8 years, range 0 - 8). The difference between subsequent timepoints is generally 1 year, give or take a few weeks to one month, depending on when the patient came in for re-evaluation, but we coded it as year 2, year 3, etc. for simplicity. For studies with missing data, longitudinal analysis with linear mixed effect model would be more advantageous than repeated measures ANOVA *(Krueger C, Tian L, 2004)*. The latter can only handle missing data via use listwise deletion, which could reduce study power substantially. Finally, linear mixed effect model is also commonly used to compare disease progression between groups with different pattern of cognitive impairment *(Scheltens et al., 2018; Qiu et al., 2019; Rongve et al., 2016)*. We therefore respectfully wish to maintain this section as it is.

Rongve A, Soennesyn H, Skogseth R, et al. Cognitive decline in dementia with Lewy bodies: a 5-year prospective cohort study. *BMJ Open*. 2016;6(2):e010357.

Krueger C, Tian L. A comparison of the general linear mixed model and repeated measures ANOVA using a dataset with multiple missing data points. *Biol Res Nurs*. 2004;6(2):151-157.

Scheltens NME, Tijms BM, Heymans MW, et al. Prominent Non-Memory Deficits in Alzheimer's Disease Are Associated with Faster Disease Progression. *J Alzheimers Dis*. 2018;65(3):1029-1039.

Qiu Y, Jacobs DM, Messer K, Salmon DP, Feldman HH. Cognitive heterogeneity in probable Alzheimer disease: Clinical and neuropathologic features. *Neurology*. 2019;93(8):e778-e790.

- Since biomarkers are not employed in this study, I think the authors should discuss more in depth in the limitation section whether the results could simply represent different pathologies (e.g., different dementias)

R1.5. We thank the reviewer for this suggestion. We have addressed this issue in the Discussion (Limitations, line 430-466), which reads as follows:

"Finally, without evidence of AD postmortem neuropathology, as is the case with most studies in clinical settings, we cannot rule out the possibility that the results could have been partly driven by misdiagnosis, i.e. dementia due to cerebrovascular disease, frontotemporal lobar degeneration, or Lewy body disease (*Beach et al., 2012*). For instance, in comparison with AD patients, those with vascular dementia demonstrate better performance on verbal learning and worse on executive functions, while scores in tests of language, attention, and visuospatial abilites were similar between the two groups (*Looi et al., 1999*). Importantly, each type of dementia is a highly heterogeneous entity by itself, hence evaluation based on neuropsychological test profiles is challenging due to individuals with different dementias showing overlapping patterns of cognitive impairment. In this study, we attempted to include participants with AD based on careful selection from a cohort of dementia patients being followed up at our medical institution. As AD dementia remains primarily a clinical diagnosis and the sensitivity and specificity of the NIA-AA diagnostic criteria have been shown to be 71%, we believe that the likelihood of misdiagnosis remains a concern but has been minimized as best as we could (*Beach et al., 2012*). Future efforts will concentrate on acquiring the missing data from a larger cohort of patients, as well as validating the result of clustering technique across time and subsets of patients with available neuropathological examination."

Beach TG, Monsell SE, Phillips LE, Kukull W. Accuracy of the Clinical Diagnosis of Alzheimer Disease at National Institute on Aging Alzheimer Disease Centers, 2005–2010. *Journal of Neuropathology & Experimental Neurology.* 2012;71(4):266–73.

Looi JC, Sachdev PS. Differentiation of vascular dementia from AD on neuropsychological tests. *Neurology*. 1999;53(4):670–8.

**ACADEMIC EDITOR**

Journal Requirements:

When submitting your revision, we need you to address these additional requirements.

1. Please ensure that your manuscript meets PLOS ONE's style requirements, including those for file naming. The PLOS ONE style templates can be found at

https://journals.plos.org/plosone/s/file?id=wjVg/PLOSOne_formatting_sample_main_body.pdf and

https://journals.plos.org/plosone/s/file?id=ba62/PLOSOne_formatting_sample_title_authors_affiliations.pdf

We have checked and ensured that the manuscript has met your style requirements.

2. Please describe in your methods section how capacity to provide consent was determined for the participants in this study. Please also state whether your ethics committee or IRB approved this consent procedure. If you did not assess capacity to consent please briefly outline why this was not necessary in this case.

Please see our answer to your 3rd requirement.

3. Please provide additional details regarding participant consent. In the ethics statement in the Methods and online submission information, please ensure that you have specified (1) whether consent was informed and (2) what type you obtained (for instance, written or verbal, and if verbal, how it was documented and witnessed). If your study included minors, state whether you obtained consent from parents or guardians. If the need for consent was waived by the ethics committee, please include this information.

If you are reporting a retrospective study of medical records or archived samples, please ensure that you have discussed whether all data were fully anonymized before you accessed them and/or whether the IRB or ethics committee waived the requirement for informed consent. If patients provided informed written consent to have data from their medical records used in research, please include this information.

Thank you very much. We have included the following information in the manuscript:

"This is a retrospective study of medical records, all data were fully anonymized before they were accessed, and the IRB committee waived the requirement for informed consent." (line 98-100)

4. Thank you for stating the following financial disclosure:

This research was funded by the Ministry of Science and Technology, Taiwan (grant number 108-2314-B-038-053-MY3) to TTTN, CY, and MC, and supported from Taipei Medical University, Taiwan (grant number 108-FRP-02) to HH, LK, and MC.

Please state what role the funders took in the study. If the funders had no role, please state: "The funders had no role in study design, data collection and analysis, decision to publish, or preparation of the manuscript." If this statement is not correct you must amend it as needed. Please include this amended Role of Funder statement in your cover letter; we will change the online submission form on your behalf.

Thank you very much. Please help us to include the Role of Funder statement as follows:

"The funders had no role in study design, data collection and analysis, decision to publish, or preparation of the manuscript."

5. We note that you have indicated that data from this study are available upon request. PLOS only allows data to be available upon request if there are legal or ethical restrictions on sharing data publicly. For more information on unacceptable data access restrictions, please see http://journals.plos.org/plosone/s/data-availability#loc-unacceptable-data-access-restrictions.

In your revised cover letter, please address the following prompts:

a) If there are ethical or legal restrictions on sharing a de-identified data set, please explain them in detail (e.g., data contain potentially sensitive information, data are owned by a third-party organization, etc.) and who has imposed them (e.g., an ethics committee). Please also provide contact information for a data access committee, ethics committee, or other institutional body to which data requests may be sent.

b) If there are no restrictions, please upload the minimal anonymized data set necessary to replicate your study findings as either Supporting Information files or to a stable, public repository and provide us with the relevant URLs, DOIs, or accession numbers. For a list of acceptable repositories, please see http://journals.plos.org/plosone/s/data-availability#loc-recommended-repositories.

We will update your Data Availability statement on your behalf to reflect the information you provide.

The Joint Institutional Review Board of Human Research at the Taipei Medical University has restricted access to the de-identified data set used in this study. Data requests may be sent to Biomedical Technology Building, Shuang-Ho campus, No. 301, Yuantong Road, Zhonghe District, New Taipei City, ohr@tmu.edu.tw.

6. PLOS requires an ORCID iD for the corresponding author in Editorial Manager on papers submitted after December 6th, 2016. Please ensure that you have an ORCID iD and that it is validated in Editorial Manager. To do this, go to ‘Update my Information’ (in the upper left-hand corner of the main menu), and click on the Fetch/Validate link next to the ORCID field. This will take you to the ORCID site and allow you to create a new iD or authenticate a pre-existing iD in Editorial Manager. Please see the following video for instructions on linking an ORCID iD to your Editorial Manager account: https://www.youtube.com/watch?v=_xcclfuvtxQ.

Thank you very much. We have updated the ORCID iD of the study's corresponding author.

7. Please review your reference list to ensure that it is complete and correct. If you have cited papers that have been retracted, please include the rationale for doing so in the manuscript text, or remove these references and replace them with relevant current references. Any changes to the reference list should be mentioned in the rebuttal letter that accompanies your revised manuscript. If you need to cite a retracted article, indicate the article’s retracted status in the References list and also include a citation and full reference for the retraction notice.

Changes to the reference list have been mentioned in the rebuttal letter. We confirmed that no papers in our reference list have been retracted as of present.

While revising your submission, please upload your figure files to the Preflight Analysis and Conversion Engine (PACE) digital diagnostic tool, https://pacev2.apexcovantage.com/. PACE helps ensure that figures meet PLOS requirements. To use PACE, you must first register as a user. Registration is free. Then, login and navigate to the UPLOAD tab, where you will find detailed instructions on how to use the tool. If you encounter any issues or have any questions when using PACE, please email PLOS at figures@plos.org. Please note that Supporting Information files do not need this step.

Thank you very much. We have used PACE to ensure that the figures meet PLOS requirements as you requested.
